# Malaria and water resource development: the case of Gilgel-Gibe hydroelectric dam in Ethiopia

**DOI:** 10.1186/1475-2875-8-21

**Published:** 2009-01-29

**Authors:** Delenasaw Yewhalaw, Worku Legesse, Wim Van Bortel, Solomon Gebre-Selassie, Helmut Kloos, Luc Duchateau, Niko Speybroeck

**Affiliations:** 1 Department of Biology, Jimma University, Jimma, Ethiopia; 2 School of Evironmental Health, Jimma University, Jimma, Ethiopia; 3 Department of Parasitology, Institute of Tropical Medicine, Antwerp, Belgium; 4 Department of Microbiology, Immunology and Parasitology, Addis Ababa University, Addis Ababa, Ethiopia; 5 Department of Epidemiology and Biostatistics, University of California Medical Center, San Francisco, CA 94143, USA; 6 Department of Physiology and Biometrics, University of Ghent, Ghent, Belgium; 7 Department of Animal Health, Institute of Tropical Medicine, Antwerp, Belgium; 8 Public Health School, Université Catholique de Louvain, Brussels, Belgium

## Abstract

**Background:**

Ethiopia plans to increase its electricity power supply by five-fold over the next five years to fulfill the needs of its people and support the economic growth based on large hydropower dams. Building large dams for hydropower generation may increase the transmission of malaria since they transform ecosystems and create new vector breeding habitats. The aim of this study was to assess the effects of Gilgel-Gibe hydroelectric dam in Ethiopia on malaria transmission and changing levels of prevalence in children.

**Methods:**

A cross-sectional, community-based study was carried out between October and December 2005 in Jimma Zone, south-western Ethiopia, among children under 10 years of age living in three 'at-risk' villages (within 3 km from dam) and three 'control' villages (5 to 8 km from dam). The man-made Gilgel-Gibe dam is operating since 2004. Households with children less than 10 years of age were selected and children from the selected households were sampled from all the six villages. This included 1,081 children from 'at-risk' villages and 774 children from 'control' villages. Blood samples collected from children using finger prick were examined microscopically to determine malaria prevalence, density of parasitaemia and identify malarial parasite species.

**Results:**

Overall 1,855 children (905 girls and 950 boys) were surveyed. A total of 194 (10.5%) children were positive for malaria, of which, 117 (60.3%) for *Plasmodium vivax*, 76 (39.2%) for *Plasmodium falciparum *and one (0.5%) for both *P. vivax *and *P. falciparum*. A multivariate design-based analysis indicated that, while controlling for age, sex and time of data collection, children who resided in 'at-risk' villages close to the dam were more likely to have *P. vivax *infection than children who resided farther away (odds ratio (OR) = 1.63, 95% CI = 1.15, 2.32) and showed a higher OR to have *P. falciparum *infection than children who resided in 'control' villages, but this was not significant (OR = 2.40, 95% CI = 0.84, 6.88). A classification tree revealed insights in the importance of the dam as a risk factor for malaria. Assuming that the relationship between the dam and malaria is causal, 43% of the malaria occurring in children was due to living in close proximity to the dam.

**Conclusion:**

This study indicates that children living in close proximity to a man-made reservoir in Ethiopia are at higher risk of malaria compared to those living farther away. It is recommended that sound prevention and control programme be designed and implemented around the reservoir to reduce the prevalence of malaria. In this respect, in localities near large dams, health impact assessment through periodic survey of potential vectors and periodic medical screening is warranted. Moreover, strategies to mitigate predicted negative health outcomes should be integral parts in the preparation, construction and operational phases of future water resource development and management projects.

## Background

Malaria is one of the most important causes of morbidity and mortality in tropical and sub-tropical countries. It is responsible for more than one million deaths each year [1]. The estimated annual global incidence of clinical malaria is 500 million cases [2]. Recent estimates indicate that more than two billion people are exposed to malaria risk in about 100 countries. Close to 90% of all malaria infections occur in sub-Saharan Africa, where malaria causes an estimated 40% of fever episodes [3-5]. More than 90% of the deaths occur in children under five years of age in Africa [6]. Most of the infections and deaths in highly endemic areas occur in children and pregnant women, who have little access to health systems [7-9]. Malaria in children is complicated by anaemia, neurological sequels from cerebral compromise, respiratory distress and sub-optimal cognitive and behavioural development [10].

Malaria transmission varies among communities largely due to environmental factors, such as proximity to breeding sites [11]. Many water resources development and management projects result in local outbreaks of malaria and other vector-borne diseases such as schistosomiasis [12], lymphatic filariasis [13] and Japanese encephalitis [14]. These outbreaks can be attributed to an increase in the number of breeding sites for mosquitoes, an extended breeding season and longevity of mosquitoes, relocation of local populations to high-risk reservoir shorelines and the arrival of migrant populations seeking a livelihood around the newly created reservoirs [15-19].

In Ethiopia, approximately 75% of the total area is estimated to be malarious, with 68% of the total population (52 million people) being at risk of infection [16]. According to the national health services statistics, malaria is among the top 10 leading causes of morbidity [16]. Proximity to micro-dams which were constructed for small irrigation development schemes is considered as one of the risk factors for increased malaria incidence [18-20]. The actual malaria cases that occur annually throughout the country are estimated to be 4–5 million [21]. Malaria is responsible for 30–40% of outpatient visits to health facilities, 10–20% of hospital admissions and 10–40% of severe cases in children under five years of age [22]. Most transmission takes place following cessation of rains [23]. Previous studies showed that malaria was more prevalent in villages that were close to small irrigation dams than in those farther away [19,20]. Ethiopia plans to increase its electricity power supply by five-fold over the next five years based on large hydropower dams to fulfill the needs of its people and support the economic growth based on large hydropower dams [24]. Ethiopia's power security is already over 85% dependent on hydropower and could grow to over 95% depending on whether all hydropower dams under construction are commissioned. Eight hydropower dams account for over 85% of Ethiopia's existing 767 MW generating capacity. Five additional hydropower sites with a combined capacity of 3,125 MW are currently under construction. Thus, it is important to look at a variety of impacts from the reservoir as it may create health problems and diseases such as malaria, schistosomiasis and lymphatic filariasis that often increase because reservoirs provide habitat for vectors (eg. mosquitoes) and intermediate hosts (eg. snails). Such investigations will also help in planning, designing and monitoring future dams.

Gilgel-Gibe hydroelectric dam, created by impounding the water of the Gilgel-Gibe River in south-western Ethiopia, is currently the largest supply of power (184 MW) in Ethiopia and is operating since 2004. During the construction of the dam, many people were relocated upstream of the reservoir, although some still remain close to the buffer zone (500–800 m from the reservoir edge at full supply level) surrounding the lake. The location of the rural villages near the newly formed reservoir may increase malaria transmission, assuming that this reservoir contributes directly or indirectly to the presence of breeding places for malaria vectors. Studies in various African countries indicate that the flight range of different species of *Anopheles *ranges from 0.8 km (*An. funestus*) [25] to an average of 1 to 1.6 km (*An. gambiae s.s*) [26], and the maximum flight range of anopheline vector mosquitoes is about 3 km [27-29].

The current study investigates the possible effects of Gilgel-Gibe hydroelectric dam on malaria transmission and prevalence among children below the age of 10 years, focusing on the distribution of infection in relation to distance of villages from the reservoir shore. Results may further guide the development of appropriate malaria interventions for communities living around the reservoir.

## Materials and methods

### Study site and population

The study area is located 260 km south-west of the capital, Addis Ababa in Oromia Regional State, south-western Ethiopia near Gilgel-Gibe hydroelectric dam. The study area lies between latitudes 7°42'50"N and 07°53'50"N and between longitudes 37°11'22"E and 37°20'36"E, at an altitude of 1,734–1,864 m above sea level. The area has a sub-humid, warm to hot climate, receives between 1,300 and 1,800 mm of annual rainfall and has a mean annual temperature of 19°C. The main socio-economic activities of the local communities are mixed farming involving the cultivation of staple crops (maize, teff and sorghum), and cattle and small stock raising. The study villages are located in Omo-Nada, Kersa and Tiro-Afeta districts (*weredas*) and have similar settlement pattern, have access to health services and are socio-economically similar. Census results taken between August and September 2005 showed a population of 6,985 in the study villages. All the communities residing in the study villages belong to the Oromo ethnic group, which is one of the largest ethnic groups in Ethiopia. The reservoir covers an area of 62 km^2 ^and is located at an altitude of 1,671 m. There are no other permanent water bodies or impoundments other than the reservoir found around the six study villages.

### Study design

A cross-sectional house-to-house survey was conducted between October and December 2005 in six villages located around the reservoir created by the newly constructed Gilgel-Gibe hydroelectric dam. Sampling was carried out by stratified cluster survey. Three villages within 3 km of the reservoir (Dogosso, Budo and Osso) and three villages located 5–8 km from its shore (Shakamsa, Sombo and Yebo) were randomly selected and designated as 'at-risk' and 'control' villages, respectively. The selection of 'at-risk' and 'control' villages was based on the established flight range of anopheline vector mosquitoes as described elsewhere in this paper [27-29]. 1, 855 children (1,081 and 774 from 'at-risk' and 'control' villages, respectively) who had lived for at least six months in those selected villages were included in the study. Bed net distribution was not started in the study villages until the end of this study but there was malaria control activity through indoor residual spraying, using DDT and malathion, which stopped four months prior to the study in both villages.

### Parasitological investigation

A parasitological study was carried out for three months (October-December 2005) to investigate the difference in malaria prevalence between 'at-risk' and 'control' villages and to characterize malaria in the area. During the survey, socio-demographic data were collected and house-to-house visits were made each month to collect blood samples from every child less than 10 years of age and thick and thin films were prepared directly from finger prick blood samples. Blood sample collection, preparation, staining technique and microscopic identification of *Plasmodium *species were performed as per standard methods [30]. The thick film served to confirm the presence or absence of the parasite, whereas the thin film was to identify the *Plasmodium *species. The initial thick films were considered negative if no parasites were seen in at least 100 oil-immersion fields of the thick film [31]. For positive slides, species and presence or absence of gametocytes was recorded. All blood films were initially read on site or at Omo-Nada District Health Center Laboratory by trained laboratory technicians. Films positive for parasites and a 10% sample of films negative for parasites were subsequently re-examined by an independent senior technician at Jimma University Specialized Hospital Laboratory. The senior microscopist was blinded to the previous microscopy results. The parasite density was counted per 300 leukocytes and was then expressed as number of parasites per microliter by assuming an average leukocyte concentration of 8,000 leukocytes/μl [32]. All *Plasmodium *positive children were treated according to the national malaria treatment guideline of the Government of Ethiopia [33].

### Statistical methods

Data were entered in and analysed with the statistical programme STATA 10 software package (StataCorp, Texas, USA). Prevalence rates were calculated from monthly positive cases. The prevalence of *Plasmodium falciparum *and *Plasmodium vivax *was calculated across age, village of residence and month of infection. Logistic regressions were conducted to check for any significant differences in the proportions of malaria cases between 'at-risk' and 'control' communities both in a univariate manner and controlling for age, sex and month. The clustering at village level was taken into account in the logistic regression models (univariate as well as multivariate) by using a marginal model with the Taylor series linearization method for estimating the variances.

### Classification tree

To investigate the potential complex interactions between the different determinants in explaining the presence of the parasite, classification trees (CART) were used [34]. This technique can be used to investigate how the available determinants can be used in creating homogenous subgroups, with either high or low prevalences. CART models are fitted by binary recursive partitioning of a multidimensional covariate space, in which the dataset is successively split into increasingly homogeneous subsets until a specified criterion is stratified. The minimum error tree was selected. CART provides a predictor ranking (variable importance) based on the contribution predictors make to the construction of the tree. This indicates how important the different independent variables are in determining the division. Importance is determined by playing a role in the tree, either as a main splitter or as a surrogate. Variable importance, for a particular predictor, is the sum across all nodes in the tree of the improvement scores that the predictor has when it acts as a primary or surrogate splitter. It is thus possible that a variable enters the tree as the top surrogate splitter in many nodes, but never as the primary splitter. Such a surrogate splitter will turn out as very important in the variable importance ranking provided by CART. More details on this technique can be found in [35].

### Prevalence fraction

The cross-sectional study allows to compute a prevalence ratio (PR) which is computed as follows: p(D+|E+)/p(D+|E-) with p a probability, D+: positive case, E+: living close to the dam and E-: living away from the dam. The 'prevalence fraction (exposed)' was calculated using the relation that PrFe = (PR-1)/PR. The PrFe expresses the proportion of disease in exposed individuals that is due to the exposure, assuming that the relationship is causal. Alternatively, the indicator can be viewed as the proportion of infections in the exposed group that would be avoided if the exposure were removed.

### Ethical considerations

Ethical approval for this study was obtained from Jimma University Research and Ethics Committee. Communal consent was first obtained then children were recruited after informed oral consent was sought from their parents or guardians of each child before a child was enrolled in the study.

## Results

Of the 1,855 children below the age of 10 years examined in this study, 905 (48.8%) were girls and 950 (51.2%) were boys. The mean age of children was 4.7 years and the number of children surveyed from 'at-risk' and 'control' communities was 1,081 (58.3%) and 774 (41.7%), respectively. Of the children in 'at-risk' communities, 528 (48.8%) were boys and 553 (51.2%) girls while in 'control' communities, 377 (48.7%) were boys and 397 (51.3%) were girls. Overall, 194 (10.5%) of the sampled children were positive for malaria, of which, 117 (60.3%) were positive for *P. vivax*, 76 (39.2%) for *P. falciparum *and one (0.5%) for both *P. vivax *and *P. falciparum*.

Highest *P. vivax *(60.7%) and *P. falciparum *(57.9%) positivity rates were observed in October. The *P. vivax *prevalence varied from 5.9% in children <1 year of age to 6.4% in those 5–9 years of age. The *P. falciparum *prevalence varied from 4.2% in children <1 year of age to 3.8% in those 5–9 years of age.

**Figure 1:**
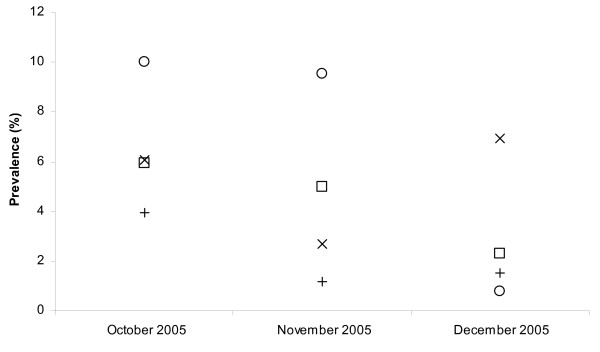
**Prevalence rates for *P. vivax *in 'at-risk' (squares) and 'control' villages (circles) and for *P. falciparum *in 'at-risk' (x-signs) and 'control' villages (+-signs)**.

Table 1 shows demographic, distance and temporal relationships with malaria infection. The monthly *P. vivax *point prevalence during the three months ranged from 0.8% to 10.0% and form 2.3% to 5.9% in 'at-risk' and 'control' villages, respectively. Monthly *P. falciparum *point prevalence during the three months ranged from 2.7% to 6.9% and from 1.2% to 4.0% in 'at-risk' and 'control' villages, respectively (Table 1). The peak prevalence rate for *P. vivax *was observed in October and gradually decreased during November to December, while the prevalence rate for *P. falciparum *showed a late increase in December (Figure 1).

**Table 1:** Demographic, distance and temporal relationships with malaria infection, *Plasmodium vivax *(Pv) and *Plasmodium falciparum *(Pf), in Gilgel-Gibe dam area, south-western Ethiopia, 2005

	Pv		Pf	
Variable	Rate	Crude OR (95% CI)	Rate	Crude OR (95% CI)

Age (years)				
<1 'control'	4/96 (4.2%)	1	3/96 (3.1%)	1
'at-risk'	13/190 (6.8%)	1.69 (0.52,5.52)	9/190 (4.7%)	1.54 (0.49,4.79)
1–4 'control'	18/396 (4.5%)	1	13/396 (3.3%)	1
'at-risk'	34/429 (7.9%)	1.81 (1.21,2.71)**	23/429 (5.4%)	1.67 (1.42,6.66)
5–9 'control'	12/282 (4.3%)	1	1/282 (0.4%)	1
'at-risk'	36/462 (7.8%)	1.9 (0.76,4.77)	27/462 (5.8%)	17.4 (1.22,249.24)**
Village/groups				
'control'	34/774 (4.4%)	1	17/774 (5.4%)	1
'at-risk'	83/1081 (7.7%)	1.81 (1.17,2.79)**	59/1081 (2.2%)	2.57 (1.01,6.57)**
Month				
October 'control'	15/253 (5.9%)	1	10/253 (4.0%)	1
'at-risk'	56/559 (10.0%)	1.76 (0.88,3.53)*	34/559 (6.1%)	1.57 (0.32,7.71)
November 'control'	13/260 (5.0%)	1	3/260 (1.2%)	1
'at-risk'	25/262 (9.5%)	2.00 (1.38,2.92)**	7/262 (2.7%)	2.35 (0.17,32.73)
December 'control'	6/261 (2.3%)	1	4/261 (1.5%)	1
'at-risk'	2/260 (0.8%)	0.33 (0.02,4.96)	18/260 (6.9%)	4.78 (1.03,22.23) **

* = significant at 0.1 level ** = significant at 0.05 level

The *P. vivax *prevalence was significantly higher in 'at-risk' communities compared to the 'control' communities in November (OR = 2.00, 95% CI = 1.38, 2.92) and the *P. falciparum *prevalence was significantly higher in 'at-risk' communities in December (OR = 4.78, 95% CI = 1.03, 22.23) (Table 1). Differences between the two communities in malaria globally (*P. vivax *and *P. falciparum *together) were obvious and statistically significant in all months (p < 0.01).

In general, significantly higher malaria prevalence was observed in children living within 3 km from the reservoir than those living farther away (OR = 1.81, 95% CI = 1.17, 2.79 for *P. vivax *and OR = 2.57, 95% CI = 1.01, 6.57 for *P. falciparum*) (Table 1). *P. vivax *prevalence rates differed significantly between 'at-risk' and 'control' communities among children 1–4 years of age (OR = 1.81, 95% CI = 1.21, 2.71) and *P. falciparum *prevalence rates differed significantly between 'at risk' and 'control' communities among 5–9 years of age (OR = 17.4, 95% CI = 1.22, 249.24).

Moreover, in a multivariate analysis controlling for age, sex and time of data collection, it appeared that children who resided in 'at-risk' villages close to the dam were more likely to have a *P. vivax *infection than children who resided in 'control' villages (OR = 1.63, 95% CI = 1.15, 2.32) (Table 2). The results in Table 2 further indicate a *P. vivax *infection difference between boys and girls, however this was not significant at the 0.05 level (p = 0.054). The multivariate analysis indicated that, while controlling for age, sex and time of data collection, children who resided in 'at-risk' villages close to the dam had a higher OR to have *P. falciparum *infection than children who resided in 'control' villages but this was not significant at the 0.05 level (OR = 2.40, 95% CI = 0.84, 6.88). Finally, while controlling for age, sex and time of data collection, children who resided in 'at-risk' villages close to the dam were at a higher risk to have a *Plasmodium *infection (*P. falciparum *and *P. vivax *combined) than children who resided in 'control' villages (OR = 1.97, 95% CI = 1.24, 3.12) (Table 2).

**Table 2:** Adjusted odds ratios (ORs) using a design-based logistic regression of malaria infection for *Plasmodium vivax *(Pv) and *Plasmodium falciparum *(Pf) by age, gender, month and village of residence in Gilgel-Gibe dam area, south-western Ethiopia, 2005.

Variable		Adjusted OR Pv	p-value	Adjusted OR Pf	p-value	Adjusted OR *Plasmodium *postivity	p-value
Village	'at risk'	1.63	0.015 **	2.40	0.085 *	1.97	0.013**
	'control'	1.00	--	1.00.	--	1.00	--

Month	October	1.00	--	1.00	--	1.00	--
	November	0.88	0.428	0.39	0.199	0.68	0.200
	December	0.18	0.096 *	0.89	0.828	0.41	0.062*

Age (yrs)	<1	1.00	--	1.00	--	1.00	--
	1–4	1.19	0.209	1.17	0.241	1.20	0.083*
	5–9	1.15	0.710	.94	0.890	1.08	0.837

Sex	Male	1.00	--	1.00	--	1.00	--
	Female	1.79	0.054	0.87	0.541	1.33	0.146

* = significant at 0.1 level ** = significant at 0.05 level

**Figure 2:**
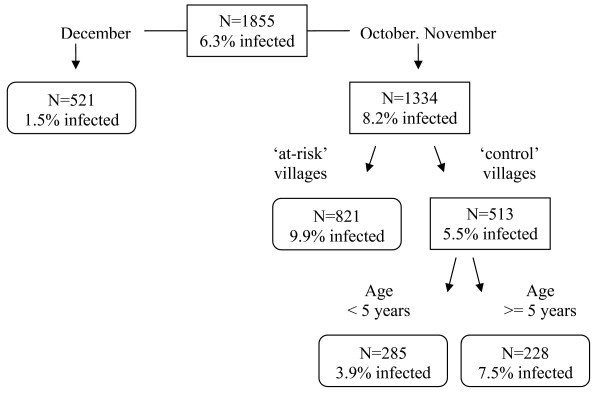
**Classification tree of the risk factors for *P. vivax *infection**.

Figure 2 shows the classification tree for *P. vivax *reproduced by CART. The children are first split into two groups: those sampled in December (prevalence = 1.5%) and those sampled in October-November (prevalence = 8.2%). The group of children sampled in October-November was further split in children living in 'at-risk' communities (prevalence = 9.9%) and those living in 'control' communities (prevalence = 5.5%). The group of children living in 'control' communities was further split in children of age below 5 years (prevalence = 3.5%) and above 5 years (prevalence = 7.5%). According to the overall discriminatory power (i.e. the relative importance) in the CART analysis, month emerged as the strongest overall discriminating risk factor for a *P. vivax *infection (Score (Sc) = 100), followed by village type (Sc = 20.21) and age (Sc = 8.73) and sex (Sc = 2.19). The classification tree corresponds well with the *P. vivax *trends in Figure 1. Indeed, the trends show that in December the *P. vivax *prevalences in 'at-risk' and 'control' communities are both low and that the difference between 'at-risk' and 'control' communities are especially clear in October-November.

**Figure 3:**
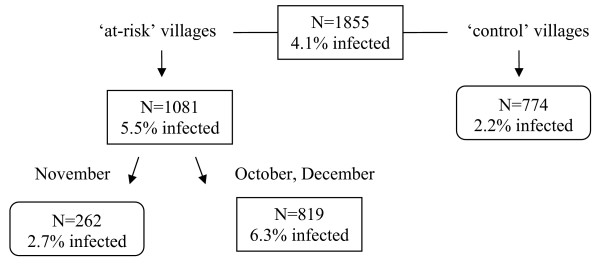
**Classification tree of the risk factors for *P. falciparum *infection**.

Figure 3 shows the classification tree for *P. falciparum *reproduced by CART. Children are first split into children living in 'at-risk' communities (prevalence = 5.5%) and those living in 'control' communities (prevalence = 2.2%). The group of children living in 'at-risk' communities was further split in children sampled in November (prevalence = 2.7%) and those sampled in October and December (prevalence = 6.3%). According to the overall discriminatory power in the CART analysis, village type emerged as the strongest overall discriminating risk factor for malaria *P. falciparum *infection (Sc = 100), followed by month (Sc = 41.3). The other variables, age and sex had a zero-Score. The classification tree corresponds well with the *P. falciparum *trends in Figure 1. The trends show that P. *falciparum *prevalences are lower in 'control' communities and that in 'at- risk' communities the prevalences were lower in November.

The 'prevalence fraction (exposed)' PrFe, for malaria (*P. vivax *and *P. falciparum *together), measuring the effect of the dam, was calculated using prevalence ratio p(D+|E+)/p(D+|E-) = 7.7/4.4 = 1.75 and is PrFe = (PR-1)/PR. = 0.43. This means that 43% of the malaria occurring in children can be attributed to the dam, assuming that the relationship is causal.

## Discussion

In this study, after controlling for age, sex and time, the prevalence of *P. vivax *(7.7%) in children who reside with in 3 km of the reservoir created by the Gilge-Gibe hydroelectric dam was significantly higher than in children living in more distant villages (4.4%) although the villages have a similar eco-topography. *Plasmodium falciparum *prevalence rates in 'at-risk' communities (5.4%) were higher than in 'control' communities (2.2%), but the difference showed no statistical significance (p-value = 0.085). Overall, the *Plasmodium *prevalence near the reservoir was statistically higher as compared to the *Plasmodium *prevalence in more distant communities (p-value = 0.013). The main reason for the higher prevalence of malaria among children living close to the reservoir may be due to the man-made ecological transformations, which may influence the presence of mosquito-breeding site and might have an impact on the behaviour, parity rate and longevity of malaria vectors of the study area. A similar study in Cameroon showed a malaria prevalence of 36% in residents living in close proximity to a man-made lake compared with a prevalence of 25% in a village 14 km away [36]. In India, a 2.4-fold increase in malaria cases and an over four-fold increase in annual parasite incidence were recorded among children in villages close to a reservoir as compared to more distant villages [37]. A high malaria prevalence, up to 47%, was recorded around the Mantali dam in Senegal, constructed to provide hydropower and irrigation, compared to prevalences of 27.3% and 29.6% in two communities downstream the dam [38]. Risk factors for malaria infections in the Gilgel-Gibe area might be proximity to the dam, as the low socioeconomic status, the health infrastructure and the malaria control methods appear to be similar in 'at-risk' and 'control' communities.

This study indicates that children between the age of one and four years tend to have a higher malaria prevalence than children below the age of one year (p-value = 0.083, non-significant for *P. vivax *and *P. falciparum *together). This could be because the older children, in contrast to younger children, spend outdoors in the evening when peak biting activities of malaria vector mosquitoes are high or the greater use of anti-malarial drugs in early childhood [39]. A similar study in Gabon showed lower malaria prevalence in children less than six months (3.7%) than in children at the age of 47 months (47.5%) [40], which was attributed to low number of children less than one year of age and immunity acquired from mothers as difference in the risk of infection among different age groups could be associated to differences in the immunological status. The risk of infection first increases with age and then decreases when the individual reaches a certain degree of immunity due to exposure to the parasite. This was indicated in the study reported in this paper as well. *Plasmodium *prevalence rates in children between one and four years, below one year and children age of 5–9 years showed no statistically significant differences.

According to several reviews, *P. falciparum *is the dominant species in Ethiopia, followed by *P. vivax*, accounting for 60% and 40% of all malaria cases, respectively [16,41]. In the present study, the predominant species was *P. vivax *followed by *P. falciparum*. *Plasmodium vivax *was found in 117 (60.3%) children, *P. falciparum *in 76 (39.2%) and mixed infection in one (0.5%) child. The other two *Plasmodium *species, *P. malariae *and *P. ovale *were absent. A similar distribution (69% *P. vivax *and 31% *P. falciparum*) was reported in a previous study [42]. But, in central Ethiopia, Woyessa *et al *[43] reported the predominance of *P. falciparum *during October while *P. vivax *tends to dominate during November. A parasitological community-based study conducted by Gebreyesus *et al *[20] on the impact of small irrigation dams on malaria burden in northern Ethiopia also revealed a predominance of *P. falciparum*. The prevalence of malaria infections varies seasonally, with *P. vivax *dominating in the dry season (March-June) and *P. falciparum *peaking in September-October, after the end of the main rainy season [16]. Hence, the proportion of malaria cases due to the two parasite species can vary across seasons and localities. Ramos *et al *[44] reported variability in the distribution of malaria parasites (22.4%–54.7% *P. vivax *and 40.9%–73.4% of *P. falciparum*) during different seasons.

The classification trees show that using this non-parametric technique allows obtaining a better insight in the data structure and the available interactions between determinants in their influence on (or relation with) malaria. This was also noted by Thang *et al *[30]. The classification tree results correspond well with the graphical trend observations, indicating that for *P. vivax*, children can be grouped according to month and children sampled in October-November showed higher prevalences even more when children were living in 'at-risk' communities (prevalence = 9.9%). For *P. falciparum*, the children living in 'at-risk' communities were grouped together because of higher prevalences. Within 'at-risk' communities especially children sampled in October and December showed a higher prevalence of 6.3%.

In conclusion, this study informs that children living in close proximity to the reservoir created by the newly constructed Gilgel-Gibe dam are at a greater risk of *Plasmodium *infection than children living further away, possibly due to the creation of new vector habitats around the lakeshore. Epidemiological studies focusing on vector dynamics and socioeconomic, demographic and health behaviour factors could be conducted to identify underlying causes of the spatial pattern of infection reported in this paper.

### Recommendations

In order to maximize the economic benefits generated by Gilgel-Gibe hydroelectric dam, it is recommended that preventive programmes against malaria and other vector-borne diseases be implemented along the periphery of the reservoir. Health Package programme, including bed net use and health education, early diagnosis and treatment, residual spraying and environmental management be implemented in an integrated way and strengthened to reduce disease burden from vector-borne diseases in communities living in close proximity to the new reservoir.

## Competing interests

The authors declare that they have no competing interests.

## Authors' contributions

DY conceptualized the study design, was involved in the coordination, supervision of data collection, data entry, cleaning, analysis, and drafted the manuscript; WL was involved in the design of the survey and reviewed the manuscript; WVB contributed to the discussion and critically reviewed the manuscript; SG was involved in the supervision of the laboratory work and in drafting the manuscript; HK contributed to the study design, and reviewed the manuscript; LD was involved in the interpretation of the statistical analysis and reviewed the manuscript; NS performed the statistical analysis, interpretation and was involved in drafting and revising the manuscript. All authors read and approved the final manuscript
